# Supplementary material for: HIVprotI: an integrated web based platform for prediction and design of HIV proteins inhibitors
Source: J Cheminform. 2018 Mar 9;10:12. doi: 10.1186/s13321-018-0266-y (PMC5845081; doi:10.1186/s13321-018-0266-y)
Supplement: Supplementary file 2 — Additional file 2. Source code of HIVProtI web server. [file 13321_2018_266_MOESM2_ESM.zip › HIVprotI_Source-code/hivprotI/weka-3-6-6/doc/weka/core/matrix/Matrix.html]

Matrix


---


|  |  |  |
| --- | --- | --- |
| **Overview** \| **Package** \| **Class** \| **Tree** \| **Deprecated** \| **Index** \| **Help** \| **Weka's home** | |  |
| **PREV CLASS**   **NEXT CLASS** | **FRAMES**    **NO FRAMES**     **All Classes** |  |
| SUMMARY: NESTED \| FIELD \| CONSTR \| METHOD | DETAIL: FIELD \| CONSTR \| METHOD |  |


---


## weka.core.matrix Class Matrix

```
java.lang.Object
  weka.core.matrix.Matrix
```

**All Implemented Interfaces:**: java.io.Serializable, java.lang.Cloneable, RevisionHandler

**Direct Known Subclasses:**: PaceMatrix

---

``` public class Matrix extends java.lang.Object implements java.lang.Cloneable, java.io.Serializable, RevisionHandler ```

Jama = Java Matrix class.

The Java Matrix Class provides the fundamental operations of numerical linear
algebra. Various constructors create Matrices from two dimensional arrays of
double precision floating point numbers. Various "gets" and "sets" provide
access to submatrices and matrix elements. Several methods implement basic
matrix arithmetic, including matrix addition and multiplication, matrix
norms, and element-by-element array operations. Methods for reading and
printing matrices are also included. All the operations in this version of
the Matrix Class involve real matrices. Complex matrices may be handled in a
future version.

Five fundamental matrix decompositions, which consist of pairs or triples of
matrices, permutation vectors, and the like, produce results in five
decomposition classes. These decompositions are accessed by the Matrix class
to compute solutions of simultaneous linear equations, determinants, inverses
and other matrix functions. The five decompositions are:

- Cholesky Decomposition of symmetric, positive definite matrices.- LU Decomposition of rectangular matrices.- QR Decomposition of rectangular matrices.- Singular Value Decomposition of rectangular matrices.- Eigenvalue Decomposition of both symmetric and nonsymmetric square matrices.

**Example of use:**

:   Solve a linear system A x = b and compute the residual norm, ||b - A x||.

    ```
           double[][] vals = {{1.,2.,3},{4.,5.,6.},{7.,8.,10.}};
           Matrix A = new Matrix(vals);
           Matrix b = Matrix.random(3,1);
           Matrix x = A.solve(b);
           Matrix r = A.times(x).minus(b);
           double rnorm = r.normInf();
    ```

Adapted from the JAMA package. Additional methods are tagged with the
`@author` tag.

**Version:**
:   $Revision: 1.8 $

**Author:**
:   The Mathworks and NIST, Fracpete (fracpete at waikato dot ac dot nz)

**See Also:**: Serialized Form

---

**Constructor Summary**

`Matrix(double[][] A)`
  
          Construct a matrix from a 2-D array.

`Matrix(double[][] A,
int m,
int n)`
  
          Construct a matrix quickly without checking arguments.

`Matrix(double[] vals,
int m)`
  
          Construct a matrix from a one-dimensional packed array

`Matrix(int m,
int n)`
  
          Construct an m-by-n matrix of zeros.

`Matrix(int m,
int n,
double s)`
  
          Construct an m-by-n constant matrix.

`Matrix(java.io.Reader r)`
  
          Reads a matrix from a reader.


| **Method Summary** | |
| --- | --- |
| `Matrix` | `arrayLeftDivide(Matrix B)`             Element-by-element left division, C = A.\B |
| `Matrix` | `arrayLeftDivideEquals(Matrix B)`             Element-by-element left division in place, A = A.\B |
| `Matrix` | `arrayRightDivide(Matrix B)`             Element-by-element right division, C = A./B |
| `Matrix` | `arrayRightDivideEquals(Matrix B)`             Element-by-element right division in place, A = A./B |
| `Matrix` | `arrayTimes(Matrix B)`             Element-by-element multiplication, C = A.\*B |
| `Matrix` | `arrayTimesEquals(Matrix B)`             Element-by-element multiplication in place, A = A.\*B |
| `CholeskyDecomposition` | `chol()`             Cholesky Decomposition |
| `java.lang.Object` | `clone()`             Clone the Matrix object. |
| `double` | `cond()`             Matrix condition (2 norm) |
| `static Matrix` | `constructWithCopy(double[][] A)`             Construct a matrix from a copy of a 2-D array. |
| `Matrix` | `copy()`             Make a deep copy of a matrix |
| `double` | `det()`             Matrix determinant |
| `EigenvalueDecomposition` | `eig()`             Eigenvalue Decomposition |
| `double` | `get(int i, int j)`             Get a single element. |
| `double[][]` | `getArray()`             Access the internal two-dimensional array. |
| `double[][]` | `getArrayCopy()`             Copy the internal two-dimensional array. |
| `int` | `getColumnDimension()`             Get column dimension. |
| `double[]` | `getColumnPackedCopy()`             Make a one-dimensional column packed copy of the internal array. |
| `Matrix` | `getMatrix(int[] r, int[] c)`             Get a submatrix. |
| `Matrix` | `getMatrix(int[] r, int j0, int j1)`             Get a submatrix. |
| `Matrix` | `getMatrix(int i0, int i1, int[] c)`             Get a submatrix. |
| `Matrix` | `getMatrix(int i0, int i1, int j0, int j1)`             Get a submatrix. |
| `java.lang.String` | `getRevision()`             Returns the revision string. |
| `int` | `getRowDimension()`             Get row dimension. |
| `double[]` | `getRowPackedCopy()`             Make a one-dimensional row packed copy of the internal array. |
| `static Matrix` | `identity(int m, int n)`             Generate identity matrix |
| `Matrix` | `inverse()`             Matrix inverse or pseudoinverse |
| `boolean` | `isSquare()`             returns whether the matrix is a square matrix or not. |
| `boolean` | `isSymmetric()`             Returns true if the matrix is symmetric. |
| `LUDecomposition` | `lu()`             LU Decomposition |
| `static void` | `main(java.lang.String[] args)`             Main method for testing this class. |
| `Matrix` | `minus(Matrix B)`             C = A - B |
| `Matrix` | `minusEquals(Matrix B)`             A = A - B |
| `double` | `norm1()`             One norm |
| `double` | `norm2()`             Two norm |
| `double` | `normF()`             Frobenius norm |
| `double` | `normInf()`             Infinity norm |
| `static Matrix` | `parseMatlab(java.lang.String matlab)`             creates a matrix from the given Matlab string. |
| `Matrix` | `plus(Matrix B)`             C = A + B |
| `Matrix` | `plusEquals(Matrix B)`             A = A + B |
| `void` | `print(int w, int d)`             Print the matrix to stdout. |
| `void` | `print(java.text.NumberFormat format, int width)`             Print the matrix to stdout. |
| `void` | `print(java.io.PrintWriter output, int w, int d)`             Print the matrix to the output stream. |
| `void` | `print(java.io.PrintWriter output, java.text.NumberFormat format, int width)`             Print the matrix to the output stream. |
| `QRDecomposition` | `qr()`             QR Decomposition |
| `static Matrix` | `random(int m, int n)`             Generate matrix with random elements |
| `int` | `rank()`             Matrix rank |
| `static Matrix` | `read(java.io.BufferedReader input)`             Read a matrix from a stream. |
| `LinearRegression` | `regression(Matrix y, double ridge)`             Performs a (ridged) linear regression. |
| `LinearRegression` | `regression(Matrix y, double[] w, double ridge)`             Performs a weighted (ridged) linear regression. |
| `void` | `set(int i, int j, double s)`             Set a single element. |
| `void` | `setMatrix(int[] r, int[] c, Matrix X)`             Set a submatrix. |
| `void` | `setMatrix(int[] r, int j0, int j1, Matrix X)`             Set a submatrix. |
| `void` | `setMatrix(int i0, int i1, int[] c, Matrix X)`             Set a submatrix. |
| `void` | `setMatrix(int i0, int i1, int j0, int j1, Matrix X)`             Set a submatrix. |
| `Matrix` | `solve(Matrix B)`             Solve A\*X = B |
| `Matrix` | `solveTranspose(Matrix B)`             Solve X\*A = B, which is also A'\*X' = B' |
| `Matrix` | `sqrt()`             returns the square root of the matrix, i.e., X from the equation X\*X = A. |
| `SingularValueDecomposition` | `svd()`             Singular Value Decomposition |
| `Matrix` | `times(double s)`             Multiply a matrix by a scalar, C = s\*A |
| `Matrix` | `times(Matrix B)`             Linear algebraic matrix multiplication, A \* B |
| `Matrix` | `timesEquals(double s)`             Multiply a matrix by a scalar in place, A = s\*A |
| `java.lang.String` | `toMatlab()`             converts the Matrix into a single line Matlab string: matrix is enclosed by parentheses, rows are separated by semicolon and single cells by blanks, e.g., [1 2; 3 4]. |
| `java.lang.String` | `toString()`             Converts a matrix to a string. |
| `double` | `trace()`             Matrix trace. |
| `Matrix` | `transpose()`             Matrix transpose. |
| `Matrix` | `uminus()`             Unary minus |
| `void` | `write(java.io.Writer w)`             Writes out a matrix. |

**Methods inherited from class java.lang.Object**

`equals, getClass, hashCode, notify, notifyAll, wait, wait, wait`

**Constructor Detail**

### Matrix

```
public Matrix(int m,
              int n)
```

:   Construct an m-by-n matrix of zeros.

    **Parameters:**: `m` - Number of rows.: `n` - Number of colums.

---


### Matrix

```
public Matrix(int m,
              int n,
              double s)
```

:   Construct an m-by-n constant matrix.

    **Parameters:**: `m` - Number of rows.: `n` - Number of colums.: `s` - Fill the matrix with this scalar value.

---


### Matrix

```
public Matrix(double[][] A)
```

:   Construct a matrix from a 2-D array.

    **Parameters:**: `A` - Two-dimensional array of doubles. **Throws:**: `java.lang.IllegalArgumentException` - All rows must have the same length **See Also:**: `constructWithCopy(double[][])`

---


### Matrix

```
public Matrix(double[][] A,
              int m,
              int n)
```

:   Construct a matrix quickly without checking arguments.

    **Parameters:**: `A` - Two-dimensional array of doubles.: `m` - Number of rows.: `n` - Number of colums.

---


### Matrix

```
public Matrix(double[] vals,
              int m)
```

:   Construct a matrix from a one-dimensional packed array

    **Parameters:**: `vals` - One-dimensional array of doubles, packed by columns (ala Fortran).: `m` - Number of rows. **Throws:**: `java.lang.IllegalArgumentException` - Array length must be a multiple of m.

---


### Matrix

```
public Matrix(java.io.Reader r)
       throws java.lang.Exception
```

:   Reads a matrix from a reader. The first line in the file should
    contain the number of rows and columns. Subsequent lines
    contain elements of the matrix.
    (FracPete: taken from old weka.core.Matrix class)

    **Parameters:**: `r` - the reader containing the matrix **Throws:**: `java.lang.Exception` - if an error occurs **See Also:**: `write(Writer)`


**Method Detail**

### constructWithCopy

```
public static Matrix constructWithCopy(double[][] A)
```

:   Construct a matrix from a copy of a 2-D array.

    :   **Parameters:**: `A` - Two-dimensional array of doubles. **Throws:**: `java.lang.IllegalArgumentException` - All rows must have the same length

---


### copy

```
public Matrix copy()
```

:   Make a deep copy of a matrix

---


### clone

```
public java.lang.Object clone()
```

:   Clone the Matrix object.

    :   **Overrides:**: `clone` in class `java.lang.Object`

---


### getArray

```
public double[][] getArray()
```

:   Access the internal two-dimensional array.

    :   **Returns:**: Pointer to the two-dimensional array of matrix elements.

---


### getArrayCopy

```
public double[][] getArrayCopy()
```

:   Copy the internal two-dimensional array.

    :   **Returns:**: Two-dimensional array copy of matrix elements.

---


### getColumnPackedCopy

```
public double[] getColumnPackedCopy()
```

:   Make a one-dimensional column packed copy of the internal array.

    :   **Returns:**: Matrix elements packed in a one-dimensional array by columns.

---


### getRowPackedCopy

```
public double[] getRowPackedCopy()
```

:   Make a one-dimensional row packed copy of the internal array.

    :   **Returns:**: Matrix elements packed in a one-dimensional array by rows.

---


### getRowDimension

```
public int getRowDimension()
```

:   Get row dimension.

    :   **Returns:**: m, the number of rows.

---


### getColumnDimension

```
public int getColumnDimension()
```

:   Get column dimension.

    :   **Returns:**: n, the number of columns.

---


### get

```
public double get(int i,
                  int j)
```

:   Get a single element.

    :   **Parameters:**: `i` - Row index.: `j` - Column index. **Returns:**: A(i,j) **Throws:**: `java.lang.ArrayIndexOutOfBoundsException`

---


### getMatrix

```
public Matrix getMatrix(int i0,
                        int i1,
                        int j0,
                        int j1)
```

:   Get a submatrix.

    :   **Parameters:**: `i0` - Initial row index: `i1` - Final row index: `j0` - Initial column index: `j1` - Final column index **Returns:**: A(i0:i1,j0:j1) **Throws:**: `java.lang.ArrayIndexOutOfBoundsException` - Submatrix indices

---


### getMatrix

```
public Matrix getMatrix(int[] r,
                        int[] c)
```

:   Get a submatrix.

    :   **Parameters:**: `r` - Array of row indices.: `c` - Array of column indices. **Returns:**: A(r(:),c(:)) **Throws:**: `java.lang.ArrayIndexOutOfBoundsException` - Submatrix indices

---


### getMatrix

```
public Matrix getMatrix(int i0,
                        int i1,
                        int[] c)
```

:   Get a submatrix.

    :   **Parameters:**: `i0` - Initial row index: `i1` - Final row index: `c` - Array of column indices. **Returns:**: A(i0:i1,c(:)) **Throws:**: `java.lang.ArrayIndexOutOfBoundsException` - Submatrix indices

---


### getMatrix

```
public Matrix getMatrix(int[] r,
                        int j0,
                        int j1)
```

:   Get a submatrix.

    :   **Parameters:**: `r` - Array of row indices.: `j0` - Initial column index: `j1` - Final column index **Returns:**: A(r(:),j0:j1) **Throws:**: `java.lang.ArrayIndexOutOfBoundsException` - Submatrix indices

---


### set

```
public void set(int i,
                int j,
                double s)
```

:   Set a single element.

    :   **Parameters:**: `i` - Row index.: `j` - Column index.: `s` - A(i,j). **Throws:**: `java.lang.ArrayIndexOutOfBoundsException`

---


### setMatrix

```
public void setMatrix(int i0,
                      int i1,
                      int j0,
                      int j1,
                      Matrix X)
```

:   Set a submatrix.

    :   **Parameters:**: `i0` - Initial row index: `i1` - Final row index: `j0` - Initial column index: `j1` - Final column index: `X` - A(i0:i1,j0:j1) **Throws:**: `java.lang.ArrayIndexOutOfBoundsException` - Submatrix indices

---


### setMatrix

```
public void setMatrix(int[] r,
                      int[] c,
                      Matrix X)
```

:   Set a submatrix.

    :   **Parameters:**: `r` - Array of row indices.: `c` - Array of column indices.: `X` - A(r(:),c(:)) **Throws:**: `java.lang.ArrayIndexOutOfBoundsException` - Submatrix indices

---


### setMatrix

```
public void setMatrix(int[] r,
                      int j0,
                      int j1,
                      Matrix X)
```

:   Set a submatrix.

    :   **Parameters:**: `r` - Array of row indices.: `j0` - Initial column index: `j1` - Final column index: `X` - A(r(:),j0:j1) **Throws:**: `java.lang.ArrayIndexOutOfBoundsException` - Submatrix indices

---


### setMatrix

```
public void setMatrix(int i0,
                      int i1,
                      int[] c,
                      Matrix X)
```

:   Set a submatrix.

    :   **Parameters:**: `i0` - Initial row index: `i1` - Final row index: `c` - Array of column indices.: `X` - A(i0:i1,c(:)) **Throws:**: `java.lang.ArrayIndexOutOfBoundsException` - Submatrix indices

---


### isSymmetric

```
public boolean isSymmetric()
```

:   Returns true if the matrix is symmetric.
    (FracPete: taken from old weka.core.Matrix class)

    :   **Returns:**: boolean true if matrix is symmetric.

---


### isSquare

```
public boolean isSquare()
```

:   returns whether the matrix is a square matrix or not.

    :   **Returns:**: true if the matrix is a square matrix

---


### transpose

```
public Matrix transpose()
```

:   Matrix transpose.

    :   **Returns:**: A'

---


### norm1

```
public double norm1()
```

:   One norm

    :   **Returns:**: maximum column sum.

---


### norm2

```
public double norm2()
```

:   Two norm

    :   **Returns:**: maximum singular value.

---


### normInf

```
public double normInf()
```

:   Infinity norm

    :   **Returns:**: maximum row sum.

---


### normF

```
public double normF()
```

:   Frobenius norm

    :   **Returns:**: sqrt of sum of squares of all elements.

---


### uminus

```
public Matrix uminus()
```

:   Unary minus

    :   **Returns:**: -A

---


### plus

```
public Matrix plus(Matrix B)
```

:   C = A + B

    :   **Parameters:**: `B` - another matrix **Returns:**: A + B

---


### plusEquals

```
public Matrix plusEquals(Matrix B)
```

:   A = A + B

    :   **Parameters:**: `B` - another matrix **Returns:**: A + B

---


### minus

```
public Matrix minus(Matrix B)
```

:   C = A - B

    :   **Parameters:**: `B` - another matrix **Returns:**: A - B

---


### minusEquals

```
public Matrix minusEquals(Matrix B)
```

:   A = A - B

    :   **Parameters:**: `B` - another matrix **Returns:**: A - B

---


### arrayTimes

```
public Matrix arrayTimes(Matrix B)
```

:   Element-by-element multiplication, C = A.\*B

    :   **Parameters:**: `B` - another matrix **Returns:**: A.\*B

---


### arrayTimesEquals

```
public Matrix arrayTimesEquals(Matrix B)
```

:   Element-by-element multiplication in place, A = A.\*B

    :   **Parameters:**: `B` - another matrix **Returns:**: A.\*B

---


### arrayRightDivide

```
public Matrix arrayRightDivide(Matrix B)
```

:   Element-by-element right division, C = A./B

    :   **Parameters:**: `B` - another matrix **Returns:**: A./B

---


### arrayRightDivideEquals

```
public Matrix arrayRightDivideEquals(Matrix B)
```

:   Element-by-element right division in place, A = A./B

    :   **Parameters:**: `B` - another matrix **Returns:**: A./B

---


### arrayLeftDivide

```
public Matrix arrayLeftDivide(Matrix B)
```

:   Element-by-element left division, C = A.\B

    :   **Parameters:**: `B` - another matrix **Returns:**: A.\B

---


### arrayLeftDivideEquals

```
public Matrix arrayLeftDivideEquals(Matrix B)
```

:   Element-by-element left division in place, A = A.\B

    :   **Parameters:**: `B` - another matrix **Returns:**: A.\B

---


### times

```
public Matrix times(double s)
```

:   Multiply a matrix by a scalar, C = s\*A

    :   **Parameters:**: `s` - scalar **Returns:**: s\*A

---


### timesEquals

```
public Matrix timesEquals(double s)
```

:   Multiply a matrix by a scalar in place, A = s\*A

    :   **Parameters:**: `s` - scalar **Returns:**: replace A by s\*A

---


### times

```
public Matrix times(Matrix B)
```

:   Linear algebraic matrix multiplication, A \* B

    :   **Parameters:**: `B` - another matrix **Returns:**: Matrix product, A \* B **Throws:**: `java.lang.IllegalArgumentException` - Matrix inner dimensions must agree.

---


### lu

```
public LUDecomposition lu()
```

:   LU Decomposition

    :   **Returns:**: LUDecomposition **See Also:**: `LUDecomposition`

---


### qr

```
public QRDecomposition qr()
```

:   QR Decomposition

    :   **Returns:**: QRDecomposition **See Also:**: `QRDecomposition`

---


### chol

```
public CholeskyDecomposition chol()
```

:   Cholesky Decomposition

    :   **Returns:**: CholeskyDecomposition **See Also:**: `CholeskyDecomposition`

---


### svd

```
public SingularValueDecomposition svd()
```

:   Singular Value Decomposition

    :   **Returns:**: SingularValueDecomposition **See Also:**: `SingularValueDecomposition`

---


### eig

```
public EigenvalueDecomposition eig()
```

:   Eigenvalue Decomposition

    :   **Returns:**: EigenvalueDecomposition **See Also:**: `EigenvalueDecomposition`

---


### solve

```
public Matrix solve(Matrix B)
```

:   Solve A\*X = B

    :   **Parameters:**: `B` - right hand side **Returns:**: solution if A is square, least squares solution otherwise

---


### solveTranspose

```
public Matrix solveTranspose(Matrix B)
```

:   Solve X\*A = B, which is also A'\*X' = B'

    :   **Parameters:**: `B` - right hand side **Returns:**: solution if A is square, least squares solution otherwise.

---


### inverse

```
public Matrix inverse()
```

:   Matrix inverse or pseudoinverse

    :   **Returns:**: inverse(A) if A is square, pseudoinverse otherwise.

---


### sqrt

```
public Matrix sqrt()
```

:   returns the square root of the matrix, i.e., X from the equation
    X\*X = A.

---


### regression

```
public LinearRegression regression(Matrix y,
                                   double ridge)
```

:   Performs a (ridged) linear regression.
    (FracPete: taken from old weka.core.Matrix class)

    :   **Parameters:**: `y` - the dependent variable vector: `ridge` - the ridge parameter **Returns:**: the coefficients **Throws:**: `java.lang.IllegalArgumentException` - if not successful

---


### regression

```
public final LinearRegression regression(Matrix y,
                                         double[] w,
                                         double ridge)
```

:   Performs a weighted (ridged) linear regression.
    (FracPete: taken from old weka.core.Matrix class)

    :   **Parameters:**: `y` - the dependent variable vector: `w` - the array of data point weights: `ridge` - the ridge parameter **Returns:**: the coefficients **Throws:**: `java.lang.IllegalArgumentException` - if the wrong number of weights were provided.

---


### det

```
public double det()
```

:   Matrix determinant

    :   **Returns:**: determinant

---


### rank

```
public int rank()
```

:   Matrix rank

    :   **Returns:**: effective numerical rank, obtained from SVD.

---


### cond

```
public double cond()
```

:   Matrix condition (2 norm)

    :   **Returns:**: ratio of largest to smallest singular value.

---


### trace

```
public double trace()
```

:   Matrix trace.

    :   **Returns:**: sum of the diagonal elements.

---


### random

```
public static Matrix random(int m,
                            int n)
```

:   Generate matrix with random elements

    :   **Parameters:**: `m` - Number of rows.: `n` - Number of colums. **Returns:**: An m-by-n matrix with uniformly distributed random elements.

---


### identity

```
public static Matrix identity(int m,
                              int n)
```

:   Generate identity matrix

    :   **Parameters:**: `m` - Number of rows.: `n` - Number of colums. **Returns:**: An m-by-n matrix with ones on the diagonal and zeros elsewhere.

---


### print

```
public void print(int w,
                  int d)
```

:   Print the matrix to stdout. Line the elements up in columns
    with a Fortran-like 'Fw.d' style format.

    :   **Parameters:**: `w` - Column width.: `d` - Number of digits after the decimal.

---


### print

```
public void print(java.io.PrintWriter output,
                  int w,
                  int d)
```

:   Print the matrix to the output stream. Line the elements up in
    columns with a Fortran-like 'Fw.d' style format.

    :   **Parameters:**: `output` - Output stream.: `w` - Column width.: `d` - Number of digits after the decimal.

---


### print

```
public void print(java.text.NumberFormat format,
                  int width)
```

:   Print the matrix to stdout. Line the elements up in columns.
    Use the format object, and right justify within columns of width
    characters.
    Note that is the matrix is to be read back in, you probably will want
    to use a NumberFormat that is set to US Locale.

    :   **Parameters:**: `format` - A Formatting object for individual elements.: `width` - Field width for each column. **See Also:**: `DecimalFormat.setDecimalFormatSymbols(java.text.DecimalFormatSymbols)`

---


### print

```
public void print(java.io.PrintWriter output,
                  java.text.NumberFormat format,
                  int width)
```

:   Print the matrix to the output stream. Line the elements up in columns.
    Use the format object, and right justify within columns of width
    characters.
    Note that is the matrix is to be read back in, you probably will want
    to use a NumberFormat that is set to US Locale.

    :   **Parameters:**: `output` - the output stream.: `format` - A formatting object to format the matrix elements: `width` - Column width. **See Also:**: `DecimalFormat.setDecimalFormatSymbols(java.text.DecimalFormatSymbols)`

---


### read

```
public static Matrix read(java.io.BufferedReader input)
                   throws java.io.IOException
```

:   Read a matrix from a stream. The format is the same the print method,
    so printed matrices can be read back in (provided they were printed using
    US Locale). Elements are separated by
    whitespace, all the elements for each row appear on a single line,
    the last row is followed by a blank line.

    Note: This format differs from the one that can be read via the
    Matrix(Reader) constructor! For that format, the write(Writer) method
    is used (from the original weka.core.Matrix class).

    :   **Parameters:**: `input` - the input stream. **Throws:**: `java.io.IOException` **See Also:**: `Matrix(Reader)`, `write(Writer)`

---


### write

```
public void write(java.io.Writer w)
           throws java.lang.Exception
```

:   Writes out a matrix. The format can be read via the Matrix(Reader)
    constructor.
    (FracPete: taken from old weka.core.Matrix class)

    :   **Parameters:**: `w` - the output Writer **Throws:**: `java.lang.Exception` - if an error occurs **See Also:**: `Matrix(Reader)`

---


### toString

```
public java.lang.String toString()
```

:   Converts a matrix to a string.
    (FracPete: taken from old weka.core.Matrix class)

    :   **Overrides:**: `toString` in class `java.lang.Object`
    :   **Returns:**: the converted string

---


### toMatlab

```
public java.lang.String toMatlab()
```

:   converts the Matrix into a single line Matlab string: matrix is enclosed
    by parentheses, rows are separated by semicolon and single cells by
    blanks, e.g., [1 2; 3 4].

    :   **Returns:**: the matrix in Matlab single line format

---


### parseMatlab

```
public static Matrix parseMatlab(java.lang.String matlab)
                          throws java.lang.Exception
```

:   creates a matrix from the given Matlab string.

    :   **Parameters:**: `matlab` - the matrix in matlab format **Returns:**: the matrix represented by the given string **Throws:**: `java.lang.Exception` **See Also:**: `toMatlab()`

---


### getRevision

```
public java.lang.String getRevision()
```

:   Returns the revision string.

    :   **Specified by:**: `getRevision` in interface `RevisionHandler`
    :   **Returns:**: the revision

---


### main

```
public static void main(java.lang.String[] args)
```

:   Main method for testing this class.


---


|  |  |  |
| --- | --- | --- |
| **Overview** \| **Package** \| **Class** \| **Tree** \| **Deprecated** \| **Index** \| **Help** \| **Weka's home** | |  |
| **PREV CLASS**   **NEXT CLASS** | **FRAMES**    **NO FRAMES**     **All Classes** |  |
| SUMMARY: NESTED \| FIELD \| CONSTR \| METHOD | DETAIL: FIELD \| CONSTR \| METHOD |  |


---
